# Comparative genomics analysis and characterization of Shiga toxin-producing *Escherichia coli* O157:H7 strains reveal virulence genes, resistance genes, prophages and plasmids

**DOI:** 10.1186/s12864-023-09902-4

**Published:** 2023-12-20

**Authors:** Natalie Naidoo, Oliver T. Zishiri

**Affiliations:** School of Life Sciences, College of Agriculture, Engineering and Sciences, University of KwaZulu-Natal, Private Bag X54001, Durban, 4000 South Africa

**Keywords:** *Escherichia coli*, O157:H7, Plasmid, Resistant, Virulent, Genomic island, Prophage

## Abstract

*Escherichia coli* O157:H7 is a foodborne pathogen that has been linked to global disease outbreaks. These diseases include hemorrhagic colitis and hemolytic uremic syndrome. It is vital to know the features that make this strain pathogenic to understand the development of disease outbreaks. In the current study, a comparative genomic analysis was carried out to determine the presence of structural and functional features of O157:H7 strains obtained from 115 National Center for Biotechnology Information database. These strains of interest were analysed in the following programs: BLAST Ring Image Generator, PlasmidFinder, ResFinder, VirulenceFinder, IslandViewer 4 and PHASTER. Five strains (ECP19–198, ECP19–798, F7508, F8952, H2495) demonstrated a great homology with Sakai because of a few regions missing. Five resistant genes were identified, however, Macrolide-associated resistance gene *mdf(A)* was commonly found in all genomes. Majority of the strains (97%) were positive for 15 of the virulent genes (*espA*, *espB*, *espF*, *espJ*, *gad*, *chuA*, *eae*, *iss*, *nleA*, *nleB*, *nleC*, *ompT*, *tccP*, *terC* and *tir*). The plasmid analysis demonstrated that the IncF group was the most prevalent in the strains analysed. The prophage and genomic island analysis showed a distribution of bacteriophages and genomic islands respectively. The results indicated that structural and functional features of the many O157:H7 strains differ and may be a result of obtaining mobile genetic elements via horizontal gene transfer. Understanding the evolution of O157:H7 strains pathogenicity in terms of their structural and functional features will enable the development of detection and control of transmission strategies.

**Supplementary Information:**

The online version contains supplementary material available at 10.1186/s12864-023-09902-4.

## Introduction

Shiga toxin-producing *Escherichia coli* (STEC) are foodborne pathogens that are a major health concern due to global disease outbreaks [1, 2]. STEC cause human gastrointestinal infections/diseases such as diarrhoea, hemorrhagic colitis and hemolytic uremic syndrome [3–6]. STEC is defined by virulence factors known as Shiga toxins [4, 7]. There are two types of Shiga toxin (Stx) (Stx1 and Stx2) that are encoded by *stx* genes that are produced in STEC [8–10]. These toxins are responsible for causing cytotoxicity in host cells [10, 11]. There are several Stx subtypes that differ in their biological activity including three subtypes for Stx1 (Stx1a, Stx1c, Stx1d) and seven subtypes for Stx2 (Stx2a to Stx2g) [12, 13]. STEC attain virulence genes through horizontal gene transfer from other pathogens [14]. Additionally, pathogenicity in STEC is also a result of the adherence factor intimin, which is encoded by the *eae* gene located in the Locus of Enterocyte Effacement (LEE) pathogenicity island [15]. LEE encodes a number of genes that play a role in the attaching and effacing [15].

Treatment for infections caused by STEC is limited, however, antibiotics can be used remove pathogens at the beginning stages of the infection [16–20]. STEC pathogens in hosts and varying environments are exposed to selective pressure leading to antibiotic resistance [21]. Research has shown that STEC are resistant to the following antibiotics in livestock and humans, tetracyclines, aminoglycosides, phenicols, streptomycin, erythromycin, carbapenems, cephalosporins, sulpha drugs and β-lactams [22–25]. Antibiotic resistance occurs via intrinsic (enzymatic degradation/ modification, efflux pumps, modification target sites or reduced cell wall permeability -) or acquired (horizontal gene transfer -) mechanisms or both [21, 26, 27]. Mobile genetic elements such as plasmid have demonstrated a role in the dissemination of antimicrobial resistance [28, 29]. Plasmids in STEC strains carry both virulent factors and antibiotic resistant (single and multiple) genes in highly conserved regions [30, 31].

Whole genome sequences available of STEC have shown high diversity because of horizontal gene transfer and genomic alterations [7, 32–37]. Using comparative genomics, identification of virulence and resistant genes and associated plasmids can be achieved to track pathogenic bacteria that pose as a public health threat. In the present study, the main aim was to compare whole genome sequences from all available *Escherichia coli* (*E. coli*) O157:H7 strains to investigate potential resistance, virulence and plasmid properties to distinguish between strains. Whole genome mapping for comparative genomics between the *E. coli* O157:H7 strains and the reference genome of pathogenic *E. coli*.

## Results

### BRIG

**Fig. 1:**
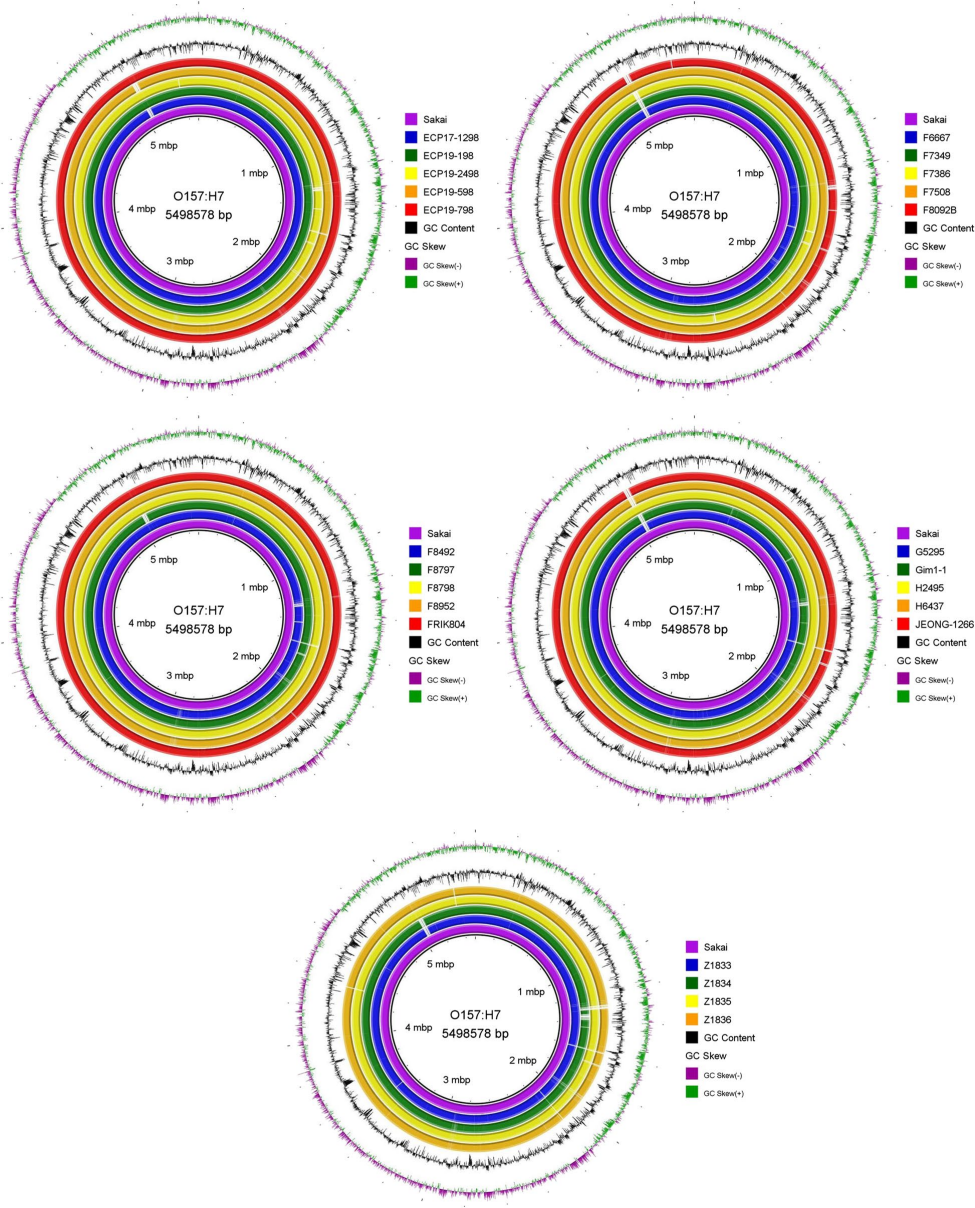
Comparison of Blast Ring Image Generated for *E. coli* O157:H7 strains

A comparative BLAST was performed using BRIG to determine homology between the reference strain (Sakai) and all other O157:H7 strains obtained. The greatest homology was observed between seven strains and Sakai: ECP19–198 (Fig. 1), ECP19–798 (Fig. 1), F7508 (Fig. 1), F8798 (Fig. 1), F8952 (Fig. 1), FRIK804 (Fig. 1) and H2495 (Fig. 1), because these strains had a few regions that were missing, and these regions were short sections of nucleotides.

### Resistance genes

**Fig. 2:**
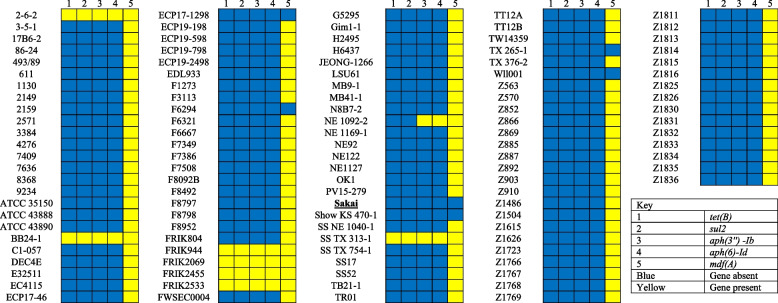
The presence of resistance genes in *Escherichia coli* O157:H7 strains

There were 5 resistance genes identified according to the ResFinder (Fig. 2): Tetracycline resistance (*tet(B)*), Sulphonamide resistance (*sul2*), Aminoglycoside resistance (*aph(3″)-Ib*), Aminoglycoside resistance (*aph(6)-Id*), Macrolide-associated resistance gene (*mdf(A)*). In strain 2–6-2, BB24–1, FRIK944, FRIK2069, FRIK2455, FRIK2533 and SS TX 313–1 all 5 resistance genes were present. In strains, ECP17–1298, F6294 Show KS 470–1, TX 265–1 and Wll001 did not have any of the resistance genes present. All other strains only had the presence of the *mdf(A)* gene except the reference strain, with the exception of the NE1092-2 strain that also had resistance to the *aph(3″)-Ib* and *aph(6)-Id* genes.

In strain 2–6-2, BB24–1, FRIK944, FRIK2069, FRIK2455, FRIK2533 and SS TX 313–1 all 5 resistance genes were present. In strains, ECP17–1298, F6294 Show KS 470–1, TX 265–1 and Wll001 did not have any of the resistance genes present. All other strains only had the presence of the *mdf(A)* gene except the reference strain.

### Plasmids

**Fig. 3:**
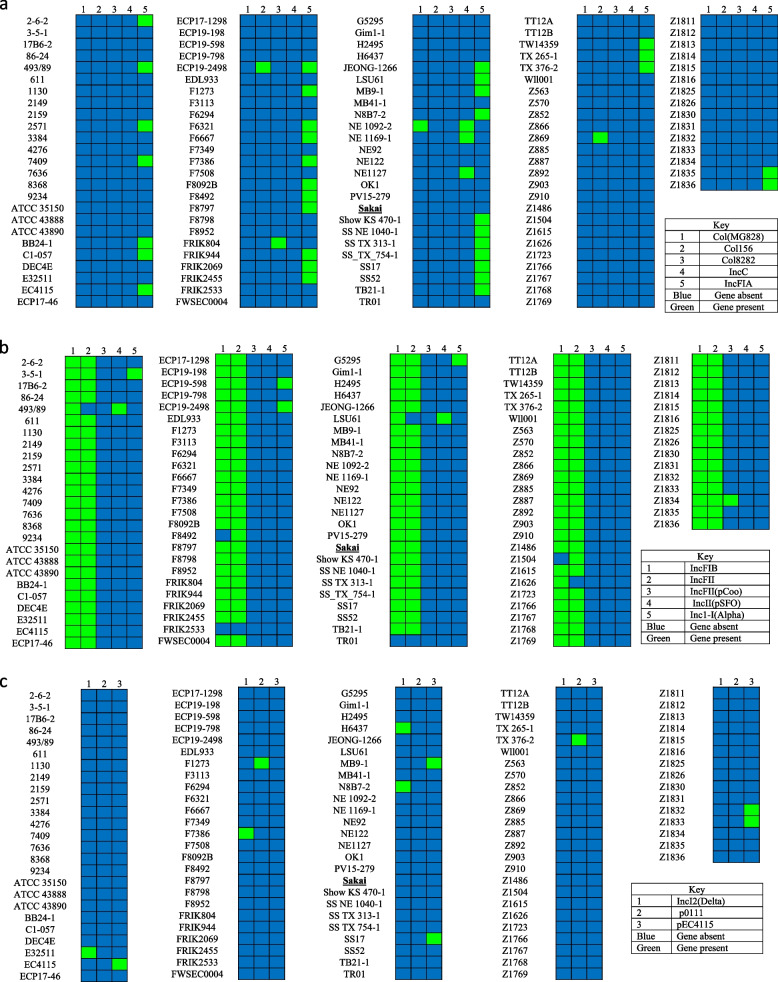
The presence of plasmids in *Escherichia coli* O157:H7 strains

There were 13 plasmids identified according to PlasmidFinder (Fig. 3): Col(MG828), Col156, Col8282, IncC, IncFIA, IncFIB, IncFII, IncFII(pCoo), IncII(pSFO), Inc1-I(Alpha), IncI2(Delta), p0111 and pEC4115. FRIK2533 and TR01 had no plasmids. The reference strain only had plasmids for IncFIB and IncFII. Col(MG828) plasmid was only present in strain NE 1092–2. Col156 was only present in Z869. Col8282 was present in FRIK804. IncC was present in NE 1092–2, NE 1169–1 and NE1127. IncFIA is present in 2–6-2, 493/89, 2571, 7409, BB24–1, C1–057, EC4115, ECP19–2498, F1273, F6321, F6667, F7386, F8092B, F8492, F8797, FRIK944, FRIK2069, FRIK2455, JEONG-1266, LSU61, MB9–1, N8B7–2, Show KS 470–1, SS NE 1040–1, SS TX 313–1, SS TX 754–1, SS17, SS52, TB21–1, TW14359, TX 265–1, TX 376–2, Z1835 and Z1836. IncFIB was present in all strains except, F8492, FRIK2533, TR01 and Z1504. IncFII was present in all except 493/89, FRIK2533, LSU61, TR01 and Z1626. IncFII(pCoo) was only present in Z1834. IncII(pSFO) was present in 493/89 and LSU61. Incl-I(Alpha) was present in 3–5-1, ECP19–598, ECP19–2498 and G5295. IncI2(Delta) was present in E32511, F7386, H6437 and N8B7–2. p0111 present in F1273 and TX 376–2. pEC4115 present in EC4115, MB9–1, SS17, Z1832 and Z1833.

### Virulence genes

**Fig. 4:**
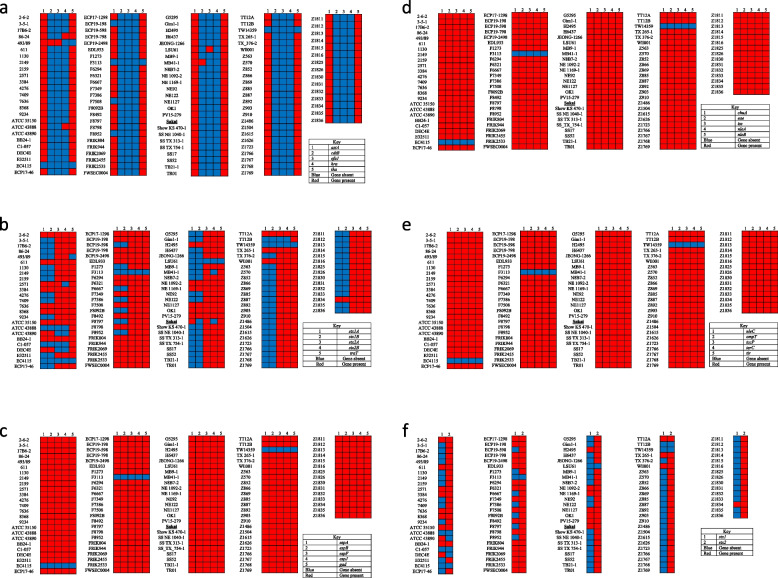
The presence of virulence genes in *Escherichia coli* O157:H7 strains

There were 27 virulence genes identified/detected by VirulenceFinder (Fig. 4): *astA, cdtB, efa1, hra, iha, stx1A, stx1B, stx2A, stxB, traT, espA, espB, espF, espJ, gad, chuA, eae, iss, nleA, nleB, nleC, ompT, tccP, terC, tir, stx1, stx2*. These virulence genes belong to categories such as, adherence, autotransporter, iron uptake, LEE-encoded TTSS effectors, non-LEE-encoded TTSS effectors, secretion system, and toxins. Virulence genes *espA, espB, espF, espJ, gad, chuA, eae, iss, nleA, nleB, nleC, ompT, tccP, terC, tir* were not present in EC4115, F3113 and TW14359 but present in all other strains. Other virulence genes that were more prevalent include: *astA* was present in all strains except EC4115, ECP19–198, F3113, F7508, F8952 and TW14359, *cdtB* was not present in all strains except 493/89, and MB41–1, *efa1* was not present in all strains except 493/89 and LSU61, *hra* was not present in all strains except 86–24 and ATCC 43888, *iha* was present in all strains except 493/89, EC4115, F3113 and TW14359, *traT* was present in all strains except 493/89, EC4115, F3113, LSU61 and TW14359. The *stx1A* was present in 38 strains and *stx1B* was present in 37 strains. The *stx2A* gene was absent in 9 strains and *stx2B* gene was absent in 12 strains. Majority of the strains did not have the *stx1* gene (only 38 strains had the gene) and the *stx2* gene was present in majority of the strains (only 9 strains did not have the gene).

### Genomic islands

The chromosomal sequences of all strains were also analysed to detect genomic islands and the locations (Table 1). Based on this analysis the number of genomic islands present ranged from 74 to 120 islands. Virulence, pathogen associated, and resistance genes were also identified. No genomic islands were identified for the following 39 strains: ECP19–198, ECP19–598, ECP19–798, ECP19–2498, TT12A, Z563, Z570, Z852, Z866, Z869, Z885, Z887, Z892, Z903, Z910, Z1486, Z1504, Z1615, Z1626, Z1723, Z1766, Z1767, Z1768, Z1769, Z1811, Z1812, Z1813, Z1814, Z1815, Z1816, Z1825, Z1826, Z1830, Z1831, Z1832, Z1833, Z1834, Z1835, Z1836. The number or virulence genes present ranged from 15 to 46. The number or pathogen associated genes ranged from 19 to 40. The number or resistant genes ranged present from 1 to 5. Strain F3113 had no resistant genes present.

**Table 1:** The presence of genomic islands in *Escherichia coli* O157:H7 strains

Strain	No. of genomic islands
Sakai (reference strain)	110
2–6-2	117
3–5-1	109
17B6–2	113
86–24	91
493–89	85
611	101
1130	84
2149	83
2159	81
2571	103
3384	96
4276	80
7409	105
7636	97
8368	91
9234	89
ATTC 35150	99
ATCC 43888	95
ATCC 43890	91
BB24–1	102
C1–057	107
DEC4E	91
E32511	101
EC4115	106
ECP17–46	104
ECP17–1298	111
EDL933	106
F1273	105
F3113	97
F6294	110
F6321	102
F6667	105
F7349	102
F7386	112
F7508	115
F8092B	99
F8492	104
F8797	120
F8798	110
F8952	100
FESEC0004	94
FRIK804	113
FRIK944	115
FRIK2069	98
FRIK2455	101
FRIK2533	106
G5295	107
Gim1–1	90
H2495	102
H6437	110
JEONG-1266	94
LSU61	96
MB9–1	98
MB41–1	97
N8B7–2	106
NE 1092–2	119
NE 1169–1	100
NE92	97
NE122	94
NE1127	103
OK1	99
PV15–279	109
Show KS 470–1	110
SS NE 1040–1	108
SS TX 313–1	113
SS TX 754–1	109
SS17	102
SS52	95
TB21–1	103
TR01	108
TT12B	95
TW14359	101
TX 265–1	108
TX 376–2	92
Wll001	92
Wll001	92

### PHASTER

Chromosomal sequences analysed by PHASTER identified phage-like elements in all 115 *E. coli* O157:H7 strains. The reference strain had 13 intact prophages, 5 questionable prophages, 4 incomplete prophages and 49.86% GC (guanine-cytosine content). The following data are significant results: Strains 1130, 2149, 2159, 4276, NE92 all had a total of 18 prophages (9 intact, 6 questionable and 3 incomplete) and percentage GC of 50.52%. Strains F7349, FWSEC004, NE 1169–1 and NE1127 had a total of 21 prophages (11 intact, 4 questionable and 6 incomplete) and percentage GC of 50.50%. Fourteen strains (Z852, Z903, Z1626, Z1766, Z1768, Z1769, Z1812, Z1815, Z1816, Z1825, Z1830, Z1831, Z1832 and Z1833) had 21 prophages (13 intact, 5 questionable and 3 incomplete) and percentage GC of 50.53%. The results for the PHASTER analysis are in Additional file 2.

## Discussion

*E. coli*, specifically strain O157:H7 has become a well-known foodborne pathogen associated with human disease because of the genome constantly changing through mutation events and horizontal gene transfer [38–43] enables strains to diverge and adapt to colonize carrier host causing diseases in humans or survive in external environments [44–47]. Hence, it is imperative to understand the genomic diversity and adaptability of the O157:H7 strains to predict severity of the disease, understand bacterial pathogenesis, identify specific biomarkers, trace origin, determine epidemiology and develop vaccines [48].

In the present study, we used comparative genomics to analyze chromosomal sequence of *E. coli* strain O157:H7 (Sakai), to determine its genetic and functional attributes to other well-characterized O157 strains. Bioinformatic tools that were available online were used to obtain information such as chromosomal homology and presence of plasmids, resistance genes, virulence genes, genomic islands, and prophages in the O157:H7 reference strain (Sakai) and other strains of interest.

BRIG was used to generate circular maps of the reference strain (Sakai) to other strains of interest and determines the chromosomal similarity of sequences There were five strains that showed a high level of similarity, namely, ECP19–198, ECP19–798, F7508, F8798, F8952, FRIK804 and H2495. This suggests that there are not many differences in the genetic make-up of these strains. Differences in the chromosomal locations of multiple O157:H7 strains can provide information on the impact that mobile elements or bacteriophages, etc. have on virulence, resistance and other aspects [41]. All *E. coli* share a core genome sequence that is approximately 4.1 Mb, however in pathogenic *E. coli* like the O157:H7 strains insertion of mobile DNA elements such as phages, genomic islands, transposons create a variability in the genome sizes among the various O157:H7 strains [41, 48–50].

In pathogenic bacteria such as *E. coli*, antimicrobial resistance genes play and integral role in becoming resistant against various drugs/medications that are used to treat diseases [51]. To achieve antibiotic resistance, entry of the antibiotic is hindered by various efflux mechanisms [51]. The *E. coli* O157:H7 strains were susceptible to five antibiotics, with the highest susceptibility against macrolide-associated resistance gene (*mdf(A)*) (95%), followed by 8 (7%) to aminoglycoside resistance (*aph(3″)-Ib*) and aminoglycoside resistance (*aph(6)-Id*) and 7 (6%) to tetracycline resistance (*tet(B)*), sulphonamide resistance (*sul2*). The key facilitator of the transport protein superfamily is the putative membrane protein (mdfA) which is coded by the *mdfA* gene and made up of 410 amino acid compounds [52]. Cationic and zwitterionic lipophilic compounds (benzalkonium, daunomycin, ethidium bromide, puromycin, rifampin, rhodamine, tetracycline and tetraphenylphosphonium) have a greater resistance to cells that express mdfA [52]. *mdfA* is also known to be resistant to vital antibiotics such as fluoroquinolones, erythromycin, chloramphenicol, and aminoglycosides [53]. From one hundred and fifteen *E. coli* O157:H7 strains evaluated, seven (6%) were resistant to all five antimicrobial resistant genes which suggests multi-drug resistant [54]. As a result, bacterial resistance increases against antibiotics since resistance in bacteria is can be obtained through bacterial gene transfer [55]. Antibiotics are used in animals for growth promotion for food consumption, in human and veterinary medicine to treat and prevent infection and control spreading of the disease [56, 57]. Thus, the overuse and negligent use of antibiotics contributes to resistance.

Most plasmids are known to have an association with antimicrobial and/or virulence resistance [58]. Among the 13 plasmids identified, the IncF group of plasmids were more prevalent. IncF plasmids systems cause autonomous replication and code for addiction systems regularly based on toxin-antitoxin factors [59]. IncF plasmids most times encode for FII together with FIA and/or FIB [60]. IncFIB and IncFII represented majority of the strains, 97 and 96% respectively, IncFIA represented 30%, IncFII(pSFO) represented 0.017% and IncFII(pCoo) represented 0.008%. The IncF incompatibility family characterizes most plasmids that are associated with virulence in *E. coli* [61]. A study by Lambrecht and others in 2018 [62], showed that the FII-FIB combination was prevalent in commensal multi drug resistant *E. coli* in farm animals. Although IncF plasmids are well adapted in *E. coli*, these plasmids have a limited host range [63, 64]. Similarly, a comparative genomics study by Noll and others in 2018 showed that almost half their sample size (44%) identified IncF plasmids [65]. However, it is important to note that the pO157 plasmid is well studied in *E. coli* O157:H7 and other plasmids that are carried are not [66]. Previous studies have shown that IncF plasmids can combine many genes that cause resistance to antimicrobials such as, aminoglycosides, β-lactams, chloramphenicol, quinolones and tetracyclines [67, 68].

The current study identified multiple virulence genes in all the O157:H7 strains. Out of the 27 virulent genes identified, 15 virulence genes (*espA, espB, espF, espJ, gad, chuA, eae, iss, nleA, nleB, nleC, ompT, tccP, terC, tir*) was found dominating in majority of the O157:H7 strains (97%). These virulent genes belong to categories such as adherence, iron uptake, toxins, Shiga toxin, non-LEE and LEE-encoded TTSS effector and secretion system. *Tir* is a T3SS effector in STEC that plays a role as the receptor for the outer membrane protein intimin which facilitates interactions between the pathogen cell and host cell to get α-actinin to the pedestal for formation of attaching and effacing intestinal lesions [69]. The *tccP* gene codes for an effector protein that plays a direct role in EHEC infection [70]. Strains become extremely pathogenic when *tccP* gene is present together with *espJ, stx1a, stx2a* intimin and *tir* [71]. Intimin facilitates intimate attachment, this is encoded by the *eae* gene which enable attaching and effacing intestinal lesions between *E. coli* O157:H7 and host cell [72]. The above-mentioned genes play an important role in making strains virulent [2] thus, the Sakai strain was used as a reference strain. *E. coli* O157:H7 strains either express *Stx1, Stx2*, or both genes, however the more toxic of the two genes is *Stx2* which causes hemorrhagic colitis and hemolytic uremic syndrome [2, 73, 74]. When isolates do not harbour the stx genes they are known as non-Shiga toxigenic *E. coli* O157:H7 [75]. A study by Iwu and others analysed O157:H7 strains from irrigation water and agricultural soil in two district municipalities in South Africa and showed that the overall prevalence of non-Shiga toxigenic *E. coli* O157:H7 was higher than STEC O157:H7 [75]. Non-Shiga toxigenic *E. coli* O157:H7 have been associated in severe diseases, however their influence as pathogens is not known [76].

The function of the genomic island of each strain is greatly dependent on the genetic makeup [77]. The genomic island results demonstrated varying number of GI. A study by Sharma and others in 2019 [48], identified 63 GI and 71 GI in the O157:H7 strains EDL933 and Sakai, respectively. However, in the present study, 106 GI and 110 GI in the O157:H7 strains EDL933 and Sakai, respectively. Genomic islands are known to display structural features that are similar, thus the difference in the number observed by Sharma and colleagues [48] and the present study could be a result of mobile elements being transferred by horizontal gene transfer [77, 78]. The genomic islands have the potential to contribute to the fitness, metabolic flexibility or increase the pathogenicity of the organisms [77]. The reference strain GI sequences can be aligned with GI sequence of interest to determine conserved GIs.

*E. coli* STEC strains are known to contain a high prophage content within the chromosome and sequences are highly variable among strains [79]. An approximation of 13–14% of the chromosome is made up of prophages in STEC O157:H7 [80, 81]. The number of predicted prophages varied greatly among the O157:H7 strains. The PHASTER analysis demonstrated the distribution of various bacteriophages. The results showed that there were three groups of strains that had the same prophages and GC percentage, suggesting that there is a high level of homology. To determine if these prophages are conserved phylogenetic and Basic Local Alignment Search Tool (BLAST) analysis can be done. In study by Weinroth and colleagues [82] demonstrated that all STEC O157:H7 showed great homology and shared three prophages. Bacteriophages that are that are similar suggest that they inhabit, adapt, and evolve from the same environment [83]. It is known that STEC genomes to possess prophages as well as integrative elements [84]. A study by in 2017 by Katani and colleagues [85] showed that prophages play an integral role in difference observed between closely related strains. This study revealed that gaining and losing genomic mobile elements cause changes in strains, for example two strains SS17 and SS52 are closely related, however, SS17 possess the phage CP-9330 and SS52 does not [85]. Phages seem to play an important part in the diversity and evolutionary aspects of *E. coli* strains such as O157:H7, therefore it is speculated that specific traits or mechanisms such as fitness and adherence can be transferred from strain to host [85]. This speculation and should be tested further using additional comparative phage characterization [85]. The genome of JEONG-1266, EC4115, and SS17 contained a total of 19 prophage regions which were highly conserved demonstrating a close evolutionary relationship [79].

## Conclusions

This study undertook a whole genome comparative analysis of *E. coli* O157:H7 isolates collected from NCBI to provide insight in the chromosomal homology, plasmids, resistance genes, virulence genes, genomic islands and prophages that are present. Our study demonstrated that although the *E. coli* O157:H7 strains belong to the same serotype group, mobile genetic elements can be transferred via horizontal gene resulting in differences between strains. Commensal strains can become pathogenic because the genetics in parts of their genome may code for virulent factor [10, 34]. STEC strains are able to adapt to multiple host conditions which provides these pathogens with the potential to expand their genomes [86]. Insight into the interactions between STEC strains and host cells will provide information on structural and functional features that result in the variation of STEC strains [2, 86]. This can be achieved by experimental confirmation to determine the evolving pathogenicity of *E. coli* O157:H7 strains which will shed light on developing strategies to detect and control the transmission of STEC in communities.

## Methods

### National Center for Biotechnology Information (NCBI) database

Reference strain sequence and all query strain sequences were selected from NCBI. In NCBI, *E. coli*, O157:H7 was searched. In the advance search all laboratory strains were excluded and only complete genomes were used. The list of strains used in this study is in Additional file 1

### BLAST Ring Image Generator (BRIG) used to construct circular chromosomal maps

Chromosomal maps were created to compare a reference bacterial strain to all query bacterial strains using BLAST Ring Image Generator (BRIG) [48] BRIG uses BLAST alignment to construct circular maps [87]. The annotated chromosome of *E. coli* O157:H7 Sakai was used as a reference for generating whole chromosomal sequence comparisons with query sequences. All default setting were used in BRIG.

### Plasmid identification

PlasmidFinder (https://cge.cbs.dtu.dk/services/PlasmidFinder/) database was used to identify the presence of plasmids in the O157:H7 strains [88, 89]. All sequences of interest were combined into one file and uploaded in the program. There are four different selection options: select database, select threshold for minimum % identity, select minimum % coverage and select you read types. Default settings were used for select threshold for minimum % identity and select minimum % coverage, 95 and 60%, respectively. Enterobacteriales was selected as database and assembled or draft genome/contigs for read type.

### Resistance identification

ResFinder (https://cge.cbs.dtu.dk/services/ResFinder/) database was used for the identification of resistant genes [89–91]. All sequences of interest were combined into one file and uploaded in the program. There are four different selection options: chromosomal point mutations, acquired antimicrobial resistance genes, select species and select you read types. Chromosomal point mutations and acquired antimicrobial resistance were selected. *E. coli* was selected as species and assembled or draft genome/contigs for read type.

### Virulent gene identification

VirulenceFinder (https://cge.cbs.dtu.dk/services/VirulenceFinder/) database was used for the identification of virulence genes [89, 92, 93]. All sequences of interest were combined into one file and uploaded in the program. There are four different selection options: select species, select threshold for % ID, select minimum length and select you read types. Default settings were used for select threshold for % ID and select minimum length, 90 and 60%, respectively. *E. coli* was selected as species and assembled or draft genome/contigs for read type.

### Genomic islands

Genomic islands (GIs) were first identified using IslandViewer 4 (https://www.pathogenomics.sfu.ca/islandviewer/browse/) [94] with the genome of *E. coli* Sakai strain as a reference. A GI was called when a prediction was made by at least one of the three methods (IslandPath-DIMOB, SIGI-HMM, and IslandPick).

### PHASTER

The presence of prophages in the chromosome of all strains were determined by downloading each FASTA file of the whole chromosomal sequence of this strain from NCBI followed by uploading the file to PHASTER [95, 96]. Prophages were identified into three groups: intact, questionable and incomplete based on the scores, a score that is > 90, a score that is 70–90) and a score that is < 70, respectively.

### Supplementary Information

**Additional file 1: Table S1. **Strain name and corresponding accession number.

**Additional file 2: Table S2: **The presence of prophages in *Escherichia coli* O157:H7 strains.

## Data Availability

The datasets used and/or analysed during the current study are available from the corresponding author on reasonable request.

## Abbreviations

BRIG: BLAST Ring Image Generator
*E. coli*: *Escherichia coli*
GC: guanine-cytosine content
GIs: Genomic islands
LEE: Locus of Enterocyte Effacement
NCBI: National Center for Biotechnology Information
Stx: Shiga toxin
STEC: Shiga toxin-producing *Escherichia coli*
