# Supplementary material for: Comparative genomics analysis and characterization of Shiga toxin-producing Escherichia coli O157:H7 strains reveal virulence genes, resistance genes, prophages and plasmids
Source: BMC Genomics. 2023 Dec 20;24:791. doi: 10.1186/s12864-023-09902-4 (PMC10731853; doi:10.1186/s12864-023-09902-4)
Supplement: Supplementary file 1 — Additional file 1: Table S1. Strain name and corresponding accession number. [file 12864_2023_9902_MOESM1_ESM.docx]

| Table S1: Strain name and corresponding accession number | | | |
| --- | --- | --- | --- |
| O157:H7 strain | Accession number | O157:H7 strain | Accession number |
| Sakai (reference) | NC_002695.2 | ECP19-598 | NZ_CP066753.1 |
| 2-6-2 | NZ_CP038419.1 | ECP19-798 | NZ_CP066756.1 |
| 3-5-1 | NZ_CP038416.1 | ECP19-2498 | NZ_CP066748.1 |
| 17B6-2 | NZ_CP038414.1 | EDL933 | NZ_CP008957.1 |
| 86-24 | NZ_CP038408.1 | F1273 | NZ_CP038376.1 |
| 493/89 | NZ_CP038412.1 | F3113 | NZ_CP038374.1 |
| 611 | NZ_CP038428.1 | F6294 | NZ_CP038372.1 |
| 1130 | NZ_CP017434.1 | F6321 | NZ_CP038369.1 |
| 2149 | NZ_CP017436.1 | F6667 | NZ_CP038366.1 |
| 2159 | NZ_CP017438.1 | F7349 | NZ_CP038363.1 |
| 2157 | NZ_CP038425.1 | F7386 | NZ_CP038360.1 |
| 3384 | NZ_CP017440.1 | F7508 | NZ_CP038357.1 |
| 4276 | NZ_CP017442.1 | F8092B | NZ_CP038355.1 |
| 7409 | NZ_CP038423.1 | F8492 | NZ_CP038282.1 |
| 7636 | NZ_CP038421.1 | F8797 | NZ_CP038353.1 |
| 8368 | NZ_CP017444.1 | F8798 | NZ_CP038351.1 |
| 9234 | NZ_CP017446.1 | F8952 | NZ_CP038349.1 |
| ATCC 35150 | NZ_CP038405.1 | FRIK804 | NZ_CP034384.1 |
| ATCC 43888 | NZ_CP041623.1 | FRIK944 | NZ_CP016625.1 |
| ATCC 43890 | NZ_CP045863.1 | FRIK2069 | NZ_CP015846.1 |
| BB24-1 | NZ_CP038402.1 | FRIK2455 | NZ_CP015843.2 |
| C1-057 | NZ_CP035366.1 | FRIK2533 | NZ_CP015842.1 |
| DEC4E | NZ_CP038398.1 | FWSEC004 | NZ_CP031913.1 |
| E32511 | NZ_CP038380.1 | G5295 | NZ_CP038346.1 |
| EC4115 | NC_011353.1 | Gim1-1 | NZ_CP038344.1 |
| ECP17-46 | NZ_CP040572.1 | H2495 | NZ_CP038342.1 |
| ECP17-1298 | NZ_CP040570.1 | H6437 | NZ_CP038339.1 |
| ECP19-198 | NZ_CP066759.1 | JEONG-1266 | NZ_CP014314.1 |

| Table S1 continued: Strain name and corresponding accession number | | | |
| --- | --- | --- | --- |
| O157:H7 strain | Accession number | O157:H7 strain | Accession number |
| LSU61 | NZ_CP038336.1 | Z885 | NZ_CP062763.1 |
| MB9-1 | NZ_CP040107.1 | Z887 | NZ_CP062761.1 |
| MB41-1 | NZ_CP039834.1 | Z892 | NZ_CP062758.1 |
| N8B7-2 | NZ_CP038333.1 | Z903 | NZ_CP062755.1 |
| NE 1092-2 | NZ_CP038328.1 | Z910 | NZ_CP062752.1 |
| NE 1169-1 | NZ_CP038324.1 | Z1486 | NZ_CP062749.1 |
| NE92 | NZ_CP038316.1 | Z1504 | NZ_CP062746.1 |
| NE122 | NZ_CP038319.1 | Z1615 | NZ_CP062744.1 |
| NE1127 | NZ_CP038321.1 | Z1626 | NZ_CP062742.1 |
| OK1 | NZ_CP038313.1 | Z1723 | NZ_CP062739.1 |
| PV15-279 | NZ_AP018488.1 | Z1766 | NZ_CP062736.1 |
| Show KS 470-1 | NZ_CP038309.1 | Z1767 | NZ_CP062733.1 |
| SS NE 1040-1 | NZ_CP038305.1 | Z1768 | NZ_CP062731.1 |
| SS TX 313-1 | NZ_CP038302.1 | Z1769 | NZ_CP062780.1 |
| SS TX 754-1 | NZ_CP038300.1 | Z1811 | NZ_CP062729.1 |
| SS17 | NZ_CP008805.1 | Z1812 | NZ_CP062727.1 |
| SS52 | NZ_CP010304.1 | Z1813 | NZ_CP062725.1 |
| TB21-1 | NZ_CP038292.1 | Z1814 | NZ_CP062723.1 |
| TR01 | NZ_CP033605.1 | Z1815 | NZ_CP062721.1 |
| TT12A | NZ_CP038496.1 | Z1816 | NZ_CP062719.1 |
| TT12B | NZ_CP038494.1 | Z1825 | NZ_CP062717.1 |
| TW14359 | NC_013008.1 | Z1826 | NZ_CP062715.1 |
| TX 265-1 | NZ_CP038290.1 | Z1830 | NZ_CP062713.1 |
| TX 376-2 | NZ_CP038287.1 | Z1831 | NZ_CP062711.1 |
| Wll001 | NZ_CP064167.1 | Z1832 | NZ_CP062708.1 |
| Z563 | NZ_CP062774.1 | Z1833 | NZ_CP062705.1 |
| Z570 | NZ_CP062771.1 | Z1834 | NZ_CP062702.1 |
| Z852 | NZ_CP062769.1 | Z1835 | NZ_CP062700.1 |
| Z866 | NZ_CP062766.1 | Z1836 | NZ_CP062778.1 |
| Z869 | NZ_CP062782.1 |  |  |
