# Supplementary material for: Comparative genomics analysis and characterization of Shiga toxin-producing Escherichia coli O157:H7 strains reveal virulence genes, resistance genes, prophages and plasmids
Source: BMC Genomics. 2023 Dec 20;24:791. doi: 10.1186/s12864-023-09902-4 (PMC10731853; doi:10.1186/s12864-023-09902-4)
Supplement: Supplementary file 2 — Additional file 2: Table S2: The presence of prophages in Escherichia coli O157:H7 strains. [file 12864_2023_9902_MOESM2_ESM.docx]

Table S2: The presence of prophages in *Escherichia coli* O157:H7 strains.

| O157:H7 strain | Intact Prophages | | Questionable Prophages | | Incomplete Prophages | | Total Prophages | | GC% |
| --- | --- | --- | --- | --- | --- | --- | --- | --- | --- |
| Sakai (reference) | 13 | | 5 | | 4 | | 22 | | 49.86 |
| 2-6-2 | 16 | | 4 | | 5 | | 25 | | 50.52 |
| 3-5-1 | 12 | | 4 | | 5 | | 21 | | 50.49 |
| 17B6-2 | 13 | | 4 | | 6 | | 23 | | 50.53 |
| 86-24 | 11 | | 5 | | 5 | | 21 | | 50.48 |
| 493/89 | 12 | | 3 | | 3 | | 18 | | 50.48 |
| 611 | 11 | | 5 | | 5 | | 21 | | 50.53 |
| 1130 | 9 | | 6 | | 3 | | 18 | | 50.52 |
| 2149 | 9 | | 6 | | 3 | | 18 | | 50.52 |
| 2159 | 9 | | 6 | | 3 | | 18 | | 50.52 |
| 2157 | 14 | | 4 | | 3 | | 21 | | 50.56 |
| 3384 | 11 | | 4 | | 5 | | 20 | | 50.52 |
| 4276 | 9 | | 6 | | 3 | | 18 | | 50.52 |
| 7409 | 14 | | 3 | | 5 | | 22 | | 50.56 |
| 7636 | 11 | | 4 | | 5 | | 20 | | 50.56 |
| 8368 | 11 | | 4 | | 5 | | 20 | | 50.52 |
| 9234 | 10 | | 6 | | 3 | | 19 | | 50.51 |
| ATCC 35150 | 11 | | 4 | | 6 | | 21 | | 50.51 |
| ATCC 43888 | 9 | | 5 | | 5 | | 19 | | 50.48 |
| ATCC 43890 | 11 | | 4 | | 3 | | 18 | | 50.55 |
| BB24-1 | 16 | | 4 | | 5 | | 25 | | 50.53 |
| C1-057 | 14 | | 3 | | 5 | | 22 | | 50.58 |
| DEC4E | 10 | | 3 | | 7 | | 21 | | 50.51 |
| E32511 | 13 | | 4 | | 5 | | 22 | | 50.48 |
| EC4115 | 15 | | 3 | | 4 | | 22 | | 50.52 |
| ECP17-46 | 13 | | 4 | | 4 | | 21 | | 50.49 |
| ECP17-1298 | 11 | | 4 | | 6 | | 21 | | 50.51 |
| ECP19-198 | 13 | | 3 | | 5 | | 21 | | 50.51 |
| O157:H7 strain | | Intact Prophages | | Questionable Prophages | | Incomplete Prophages | | Total Prophages | GC% |
| ECP19-598 | | 14 | | 4 | | 5 | | 23 | 50.53 |
| ECP19-798 | | 12 | | 3 | | 7 | | 22 | 50.52 |
| ECP19-2498 | | 17 | | 3 | | 6 | | 26 | 50.58 |
| EDL933 | | 11 | | 6 | | 6 | | 23 | 50.48 |
| F1273 | | 14 | | 3 | | 5 | | 22 | 50.58 |
| F3113 | | 10 | | 9 | | 6 | | 25 | 50.56 |
| F6294 | | 13 | | 3 | | 6 | | 22 | 50.55 |
| F6321 | | 13 | | 5 | | 4 | | 22 | 50.54 |
| F6667 | | 14 | | 5 | | 3 | | 22 | 50.51 |
| F7349 | | 11 | | 4 | | 6 | | 21 | 50.50 |
| F7386 | | 13 | | 4 | | 3 | | 20 | 50.49 |
| F7508 | | 12 | | 4 | | 5 | | 21 | 50.51 |
| F8092B | | 14 | | 5 | | 6 | | 25 | 50.55 |
| F8492 | | 15 | | 4 | | 5 | | 24 | 50.55 |
| F8797 | | 14 | | 4 | | 4 | | 22 | 50.49 |
| F8798 | | 12 | | 6 | | 7 | | 25 | 50.53 |
| F8952 | | 10 | | 4 | | 7 | | 21 | 50.51 |
| FRIK804 | | 12 | | 4 | | 7 | | 23 | 50.52 |
| FRIK944 | | 19 | | 4 | | 5 | | 28 | 50.59 |
| FRIK2069 | | 17 | | 6 | | 4 | | 27 | 50.55 |
| FRIK2455 | | 17 | | 6 | | 4 | | 27 | 50.55 |
| FRIK2533 | | 17 | | 6 | | 4 | | 27 | 50.55 |
| FWSEC004 | | 11 | | 4 | | 6 | | 21 | 50.50 |
| G5295 | | 14 | | 4 | | 5 | | 23 | 50.53 |
| Gim1-1 | | 9 | | 6 | | 3 | | 18 | 50.51 |
| H2495 | | 12 | | 4 | | 6 | | 22 | 50.53 |
| H6437 | | 12 | | 5 | | 4 | | 21 | 50.49 |
| JEONG-1266 | | 14 | | 4 | | 4 | | 22 | 50.49 |

| O157:H7 strain | Intact Prophages | Questionable Prophages | Incomplete Prophages | Total Prophages | GC% |
| --- | --- | --- | --- | --- | --- |
| LSU61 | 12 | 2 | 4 | 18 | 50.48 |
| MB9-1 | 14 | 4 | 5 | 23 | 50.54 |
| MB41-1 | 13 | 6 | 6 | 25 | 50.52 |
| N8B7-2 | 14 | 4 | 6 | 24 | 50.57 |
| NE_1092-2 | 16 | 5 | 3 | 24 | 50.55 |
| NE_1169-1 | 11 | 4 | 6 | 21 | 50.50 |
| NE92 | 9 | 6 | 3 | 18 | 50.52 |
| NE122 | 11 | 5 | 6 | 22 | 50.59 |
| NE1127 | 11 | 4 | 6 | 21 | 50.50 |
| OK1 | 11 | 4 | 7 | 22 | 50.52 |
| PV15-279 | 13 | 7 | 8 | 28 | 50.61 |
| Show_470-1 | 14 | 4 | 5 | 23 | 50.59 |
| SS_NE_1040-1 | 15 | 3 | 5 | 23 | 50.57 |
| SS_TX_313-1 | 14 | 7 | 4 | 25 | 50.53 |
| SS_TX_754-1 | 15 | 5 | 5 | 25 | 50.56 |
| SS17 | 15 | 3 | 4 | 22 | 50.50 |
| SS52 | 15 | 4 | 3 | 22 | 50.51 |
| TB21-1 | 14 | 4 | 5 | 23 | 50.55 |
| TR01 | 14 | 4 | 4 | 22 | 50.53 |
| TT12A | 14 | 5 | 4 | 21 | 50.49 |
| TT12B | 9 | 5 | 5 | 19 | 50.51 |
| TW14359 | 15 | 3 | 3 | 21 | 50.51 |
| TX_265-1 | 13 | 3 | 6 | 22 | 50.60 |
| TX_376-2 | 12 | 4 | 5 | 21 | 50.53 |
| Wll001 | 12 | 5 | 6 | 23 | 50.54 |
| Z563 | 13 | 5 | 2 | 20 | 50.54 |
| Z570 | 14 | 5 | 3 | 22 | 50.55 |
| Z852 | 13 | 5 | 3 | 21 | 50.52 |
| Z866 | 14 | 5 | 3 | 22 | 50.55 |

| O157:H7 strain | Intact Prophages | Questionable Prophages | Incomplete Prophages | Total Prophages | GC% |
| --- | --- | --- | --- | --- | --- |
| Z869 | 15 | 5 | 3 | 23 | 50.59 |
| Z885 | 15 | 4 | 2 | 21 | 50.54 |
| Z887 | 15 | 4 | 2 | 21 | 50.55 |
| Z892 | 14 | 4 | 5 | 23 | 50.53 |
| Z903 | 13 | 5 | 3 | 21 | 50.53 |
| Z910 | 12 | 5 | 3 | 20 | 50.54 |
| Z1486 | 11 | 6 | 3 | 20 | 50.53 |
| Z1504 | 13 | 4 | 4 | 21 | 50.54 |
| Z1615 | 14 | 4 | 3 | 21 | 50.53 |
| Z1626 | 13 | 5 | 3 | 21 | 50.53 |
| Z1723 | 16 | 5 | 3 | 24 | 50.51 |
| Z1766 | 13 | 5 | 3 | 21 | 50.53 |
| Z1767 | 14 | 4 | 3 | 21 | 50.53 |
| Z1768 | 13 | 5 | 3 | 21 | 50.53 |
| Z1769 | 13 | 5 | 3 | 21 | 50.53 |
| Z1811 | 13 | 5 | 4 | 22 | 50.53 |
| Z1812 | 13 | 5 | 3 | 21 | 50.53 |
| Z1813 | 14 | 5 | 3 | 22 | 50.57 |
| Z1814 | 11 | 6 | 4 | 21 | 50.54 |
| Z1815 | 13 | 5 | 3 | 21 | 50.55 |
| Z1816 | 13 | 5 | 3 | 21 | 50.55 |
| Z1825 | 13 | 5 | 3 | 21 | 50.56 |
| Z1826 | 13 | 5 | 2 | 20 | 50.56 |
| Z1830 | 13 | 5 | 3 | 21 | 50.53 |
| Z1831 | 13 | 5 | 3 | 21 | 50.53 |
| Z1832 | 13 | 5 | 3 | 21 | 50.53 |
| Z1833 | 13 | 5 | 3 | 21 | 50.53 |
| Z1834 | 12 | 4 | 6 | 22 | 50.60 |
| Z1835 | 14 | 3 | 5 | 22 | 50.54 |
| Z1836 | 15 | 3 | 5 | 23 | 50.56 |
